# Progressive Hemorrhagic Intracranial Lesions with Autoimmune Serologic Positivity in Severe Pneumococcal Meningitis

**DOI:** 10.51894/001c.164575

**Published:** 2026-07-31

**Authors:** Mubera Bebanic, Wasif Hafeez, Mohamed S. Siddique

**Affiliations:** 1 DMC - Sinai Grace

## 65

### INTRODUCTION

Streptococcus pneumoniae remains a leading cause of bacterial meningitis and invasive septicemia and carries substantial neurologic morbidity. While ischemic complications are well described, progressive hemorrhagic intracranial lesions during antimicrobial therapy are uncommon and create diagnostic uncertainty. In critically ill patients, the differential diagnosis includes septic emboli, evolving cerebritis or abscess, metastatic disease, and secondary central nervous system vasculitis. The emergence of positive autoimmune serologies during acute infection further complicates management, as escalation to immunosuppression in recent septicemia carries risk. We describe a critically ill patient with invasive pneumococcal meningitis complicated by enlarging hemorrhagic brain lesions and concurrent autoimmune serologic activation.

### CASE DESCRIPTION

A 47-year-old woman with GERD and chronic anemia presented with fever and rapidly progressive encephalopathy following upper respiratory symptoms. On admission, she was febrile, tachycardic, and obtunded with nuchal rigidity. Laboratory evaluation revealed KDIGO stage 3 acute kidney injury (creatinine 7.8 mg/dL, BUN 120 mg/dL) and high anion gap metabolic acidosis. Blood cultures grew Streptococcus pneumoniae. Cerebrospinal fluid demonstrated glucose <10 mg/dL, protein >1000 mg/dL, and neutrophilic pleocytosis, confirming bacterial meningitis. High-dose ceftriaxone was initiated. Her course was marked by multiorgan dysfunction requiring renal replacement therapy and intensive neurologic monitoring. Initial MRI revealed two hemorrhagic cortical lesions in the left frontal and parietal lobes with edema. Repeat contrast imaging demonstrated interval enlargement with rim enhancement and new bilateral T2/FLAIR hyperintense foci. MR venography showed no venous sinus thrombosis. Autoimmune testing revealed positive ANA and ANCA titers with lupus anticoagulant positivity, while complement levels remained normal. Given radiographic progression, CNS vasculitis was considered. However, clearance of bacteremia, absence of systemic vasculitic features, lesion distribution consistent with septic emboli, and stabilization with antimicrobial therapy supported severe pneumococcal meningitis complicated by hemorrhagic septic emboli rather than primary vasculitis. Immunosuppression was deferred.

### CONCLUSION

This case underscores the complexity of neurologic deterioration during invasive pneumococcal meningitis and illustrates how acute infection may trigger transient autoimmune serologic positivity. In critically ill patients, management should be guided by clinical trajectory and multidisciplinary assessment rather than isolated laboratory abnormalities. Diagnostic restraint is essential to avoid premature immunosuppression while ensuring appropriate treatment of life-threatening infection.

